# Opportunities and challenges of protein-based targeted protein degradation

**DOI:** 10.1039/d3sc02361c

**Published:** 2023-07-03

**Authors:** Fangfang Shen, Laura M. K. Dassama

**Affiliations:** a Department of Chemistry, Sarafan ChEM-H Institute, Stanford University USA dassama@stanford.edu; b Department of Microbiology & Immunology, Stanford School of Medicine USA

## Abstract

In the 20 years since the first report of a proteolysis targeting chimeric (PROTAC) molecule, targeted protein degradation (TPD) technologies have attempted to revolutionize the fields of chemical biology and biomedicine by providing exciting research opportunities and potential therapeutics. However, they primarily focus on the use of small molecules to recruit the ubiquitin proteasome system to mediate target protein degradation. This then limits protein targets to cytosolic domains with accessible and suitable small molecule binding pockets. In recent years, biologics such as proteins and nucleic acids have instead been used as binders for targeting proteins, thereby expanding the scope of TPD platforms to include secreted proteins, transmembrane proteins, and soluble but highly disordered intracellular proteins. This perspective summarizes the recent TPD platforms that utilize nanobodies, antibodies, and other proteins as binding moieties to deplete challenging targets, either through the ubiquitin proteasome system or the lysosomal degradation pathway. Importantly, the perspective also highlights opportunities and remaining challenges of current protein-based TPD technologies.

## Abstract 2

Targeted protein degradation strategies employing proteins as binders for degradation targets.

## Graphical abstract

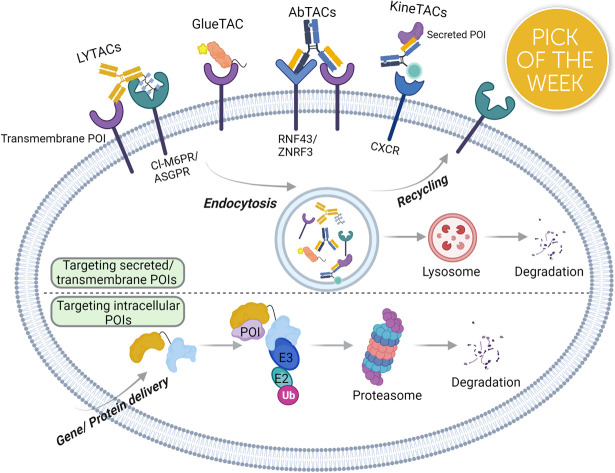

## Introduction

Recent technological advancements have greatly expanded molecular level understanding of disease pathologies, revealing a multitude of potential protein targets for therapeutic intervention.^1–5^ While traditional therapeutics such as small molecules and monoclonal antibodies are effective at blocking protein function through occupancy-driven strategies, they are often limited in targeting proteins such as transcription factors, non-enzymatic proteins, and scaffolding proteins that lack suitable binding pockets. Moreover, achieving complete protein inhibition often requires high drug dosages and continuous exposure, which can lead to increased off-target effects and resistance.^6^ Traditional targeted protein degradation (TPD) presents an exciting strategy to overcome these challenges because it functions through the depletion of proteins of interest (POIs) by inducing the interaction of cytosolic POIs and intracellular protein degradation machinery. This approach allows TPD to target difficult proteins that lack potent small-molecule inhibitors and to achieve increased efficacy at sub-stoichiometric ratios due to the catalytic nature of TPD molecules.^7^ Over the past two decades, various TPD tools such as molecular glue degraders,^8,9^ proteolysis targeting chimeras (PROTACs),^10–12^ specific and nongenetic IAP-dependent protein erasers (SNIPERs),^13^ degradation tags (dTAGs),^14,15^ autophagy targeting chimeras (AUTACs),^16^ and autophagosome-tethering compounds (ATTECs),^17^ have been developed. Encouragingly, thalidomide, a drug used in the clinic for decades, was demonstrated to function as a molecular glue degrader;^18^ other PROTACs and molecular glues have also entered clinical trials.^11,19^ All of this bodes well for the therapeutic potential of TPD platforms.

Despite these successes, challenges remain. For example, TPD platforms primarily rely on small molecule binders and the intracellular ubiquitin proteasome system (UPS), which limits their application to proteins that contain cytosolic domains and available binding pockets. In reality, transmembrane proteins, secreted proteins, and intracellular proteins that lack suitable ligand binding pockets constitute the majority of therapeutically relevant targets.^20^ Instead of using small molecules, innovative technologies have leveraged biologics such as peptides, proteins, and nucleic acids as targeting binders for challenging POIs. The first PROTAC molecule was actually a peptide-based ligand consisting of the IκBα phosphopeptide (DRHDpSGLDSM),^21^ while another peptide from hypoxia-inducible factor 1 subunit-α (HIF1α) was also frequently utilized as binder for the E3 ligase von Hippel-Lindau (VHL).^22,23^ Recently, more peptide-based PROTACs have been shown to successfully induce the degradation of proteins that include Akt,^24^ Tau,^25^ α-synuclein,^26^ PI3K/FRS2α^27^ and X-protein.^28^ Nucleic acids have also been used as binders to develop TPD systems such as transcription factor targeting chimeras (TRAFTACs),^29^ oligonucleotide-based PROTACs (O'PROTACs)^30^ and transcription factor PROTACs.^31^ There are also RNA-PROTACs for RNA binding proteins,^32^ G4-PROTACs for G4 binding proteins,^33^ and aptamer-based PROTACs.^34^ Also recently, LYTACs,^35,36^ AbTACs,^37^ PROTABs^38^ and KineTACs,^39^ which all use antibodies or nanobodies as POI binders, employed the lysosome to enable the targeted degradation of extracellular and transmembrane proteins. Even with these recent technologies, one major hurdle remained: use of biologics is largely restricted to extracellular or transmembrane proteins, as biologics lack the ability to permeate cells. We recently demonstrated the degradation of a traditionally “undruggable” intracellular POI using a cell-permeant nanobody-based degrader; that work describes one way in which this last major hurdle might be surmounted.^40^

In this Perspective, we review the progress made in protein-based degrader systems, focusing on two key aspects: (1) an overview of recent developments to deplete extracellular or transmembrane proteins using LYTACs, GluTACs, AbTACs, PROTABs, and KineTACs, in order to highlight the unique design enabling the uptake of extracellular or transmembrane proteins and facilitating access to the cellular degradation machinery; and (2) a summary of intracellular protein degraders comprising nanobodies, antibodies, or other proteins as POI and degradation machinery binders. For this aspect, we cover the identification and engineering of antibody/nanobody binders for various POIs, the degrader design strategy and protein delivery methods. We also discuss remaining challenges of protein-based degraders, such as unknown pharmacokinetics and the absence of informed design. Throughout this Perspective, we offer an extensive examination of the latest advancements in protein-based degraders, while further enhancing our understanding of essential biological factors that govern degradation processes. Moreover, we also provide insights into the potential for strategically designing these types of degraders for use in therapeutic applications and in exploring biological pathways.

## Fundamentals of TPD

**Fig. 1:**
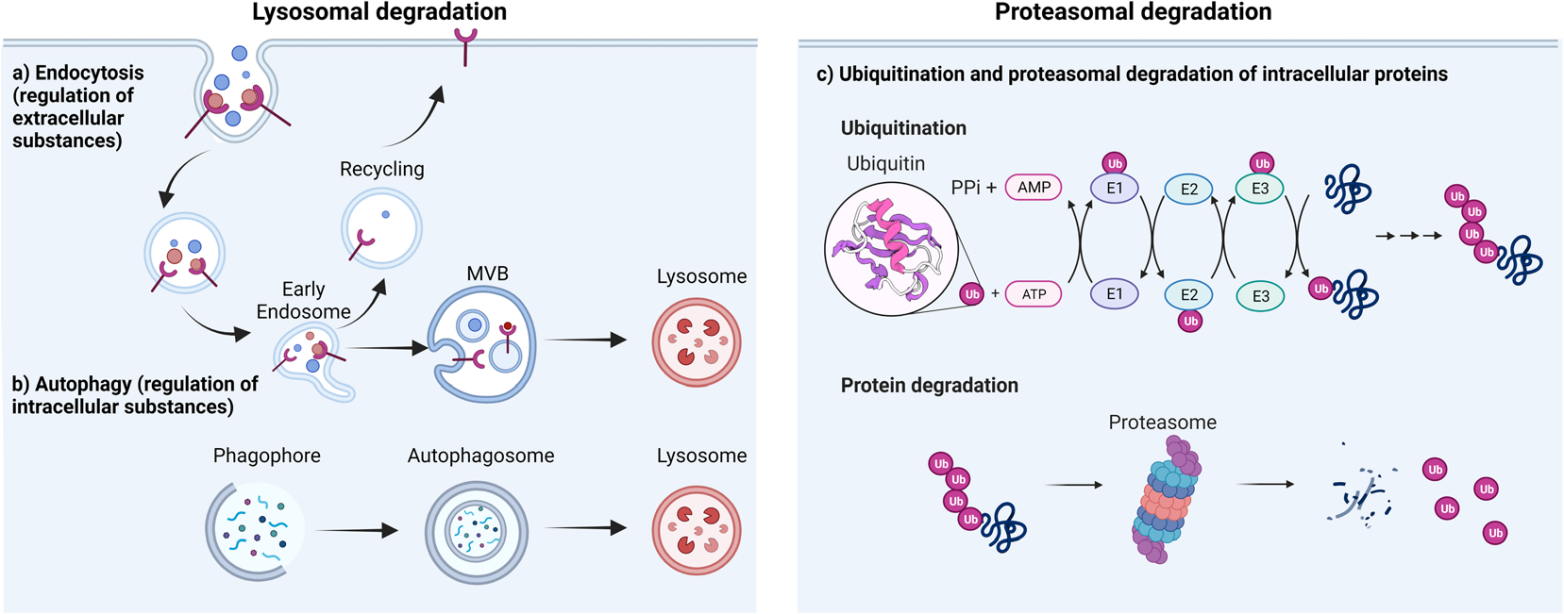
Major cellular platforms for protein degradation. Degradation of (a) extracellular substances through endocytosis, (b) intracellular substances through autophagosome/lysosome fusion and (c) intracellular substances through proteasomal pathway.

There are two major types of degradation systems in cells: the lysosomal pathway and the proteasomal pathway.^41,42^ In the lysosomal pathway, lysosomes degrade both extracellular and intracellular substances (proteins, nucleic acids, polysaccharides, and others).^43^ The extracellular substances and plasma membrane components (such as receptors or channels) enter the cell through the endocytic pathway^44^ and become degraded in the highly acidic endosome/lysosome.^45^ This contributes to plasma membrane repair, immune response, bone resorption, and pathogen elimination.^46^ Some of the internalization process is highly specific, which allows cells to efficiently internalize specific substances while ignoring others. For instance, in receptor-mediated endocytosis, cells selectively take up substances (*e.g.*, extracellular proteins) that bind to specific receptors on the cell membrane.^47^ This binding causes a conformational change in the receptor, which in turn triggers the formation of a vesicle containing the ligand–receptor complex.^48^ The vesicle then travels to the early endosome, where many endocytosed substances dissociate from their receptors in the mildly acidic compartment and are selectively sorted *via* transvesicular compartments (like multivesicular bodies, MVB, or endosomal carrier vesicles, ECVs)^49^ to fuse with the lysosome for subsequent degradation by acidic hydrolases in that compartment. Meanwhile, upon the release of substances, the receptors are recycled back to the plasma membrane through recycling endosomes (Fig. 1a). By harnessing the receptor-mediated endocytic pathway for lysosomal degradation, LYTACs have been used to degrade extracellular or transmembrane proteins.^35^ Lysosomes are also responsible for degrading intracellular damaged proteins or organelles through autophagy; autophagy allows the wrapping of damaged proteins by autophagosomes.^50^ Upon fusion of autophagosomes and lysosomes, autolysosomes are formed to degrade the cargo and release the breakdown products (amino acids) into the cytosol for reuse (Fig. 1b). The regulation of intracellular substance levels through lysosomes is of great significance for maintaining the normal metabolic activities of cells and for defending against microbial infections.^51^

The second major degradation system is the proteasomal system. In this system, proteins are degraded by the 26S proteasome, a complex of proteolytic enzymes that break down proteins into small peptides.^52^ This system has three main components: the proteasome, various ubiquitin ligases, and multiple ubiquitinating or de-ubiquitinating enzymes. Ubiquitin is a regulatory protein that is highly conserved across all forms of life. It consists of 76 amino acids with a molecular weight of 8.5 kDa. It is linked to proteins that are targeted for degradation through a multistep process called ubiquitination. Ubiquitination involves the action of three enzymes to mediate the covalent modification of proteins. In the first step, an E1 (ubiquitin activating enzyme) binds to the ubiquitin molecule through its active site. In the next step, ubiquitin is transferred to the active site of E2 (ubiquitin conjugating enzyme). In the third step, E3 (ubiquitin ligase enzyme) recognizes the substrate protein to be degraded and catalyzes the transfer of ubiquitin from the E2 protein to the substrate (Fig. 1c).

There are different types of E3 ligases, including the really interesting new gene (RING) E3s, homologous to E6-AP C terminus (HECT) E3s, and RING-between-RING (RBR) E3s.^53^ The mechanism of substrate ubiquitination differs among each type.^54^ The RING E3 ligases, most notably the Cullin–RING ligase (CRL) multi-subunit superfamily, comprise the largest known class of ubiquitin ligases.^55,56^ Human cells express seven different cullins (CUL1, 2, 3, 4A, 4B, 5 and 7) that each nucleate a multisubunit ubiquitin ligase.^57^ CUL1 CRLs, which are known commonly as SKP1, CUL1, and F-box (SCF) proteins, recruit substrates through the adaptor protein SKP1 and an F-box-protein substrate receptor;^58^ CUL2 CRLs such as VHL, and CUL5 CRLs recruit substrates through an Elongin-BC adaptor and a suppressor of cytokine signaling/Elongin-BC (SOCS/BC)-box-protein substrate receptor;^59^ CUL3 CRLs, such as speckle type BTB/POZ protein (SPOP), recruit substrates through BTB-domain-containing substrate-receptor proteins;^60^ CUL4A CRLs, which include DCAF, recruit substrates through the adaptor protein DNA-damage-binding protein-1 (DDB1).^61^ HECT E3 ligases first form a thioester intermediate with ubiquitin, then subsequently transfer it to the substrate.^62^ RBR E3 ligases use one RING domain to bind the ubiquitin loaded E2, then transfer ubiquitin to the second RING domain, which facilitates ubiquitin ligation to the substrate.^57^ In all cases, the substrate specificity of the system is determined by the E3 ligase, which binds directly to the target substrate. Following the initial ubiquitination event, the substrate-linked ubiquitin can be ubiquitinated on one of several lysine residues, leading to the formation of polyubiquitin chains.^63^

Ubiquitin has several lysine residues (K6, K11, K27, K29, K33, K48 and K63) and the free amino group located at its N-terminus that are involved in the formation of polyubiquitin linkages. Different homo- and heterotypic linkages are employed during the modification of target proteins to subsequently induce different cellular functions. For example, K63-linked ubiquitination serves as a docking site to mediate protein–protein interactions or conformational changes while the K48-linked polyubiquitin chains are involved in proteasomal degradation.^64,65^ The proteasomal degradation pathway plays a central role in removing abnormal or misfolded proteins and in regulating a variety of protein functions that allow control of the physiological behavior of the cell. Small molecule PROTACs, molecular glue degraders, and protein-based degraders for intracellular POIs all primarily utilize this pathway to induce protein degradation.

As the two most significant degradation pathways in cells, the proteasomal pathway and the lysosomal system independently regulate the degradation of substances in cells and interact with each other to achieve compensatory or synergistic outcomes. By harnessing the power of these two degradation systems, TPD technologies mediate the depletion of both intracellular, transmembrane, and extracellular proteins with high specificity and efficiency.

## Protein-based degraders that target extracellular or transmembrane proteins

Extracellular and transmembrane proteins constitute 40% of all protein-encoding genes and comprise those that are critical in cancer, ageing-related diseases, and autoimmune disorders.^20^ However, small molecule PROTACs and molecular glue degraders rely on the intracellular protein degradation machinery, which limits their use to proteins with intracellular domains. To deplete extracellular or transmembrane proteins, new strategies by employing engineered antibodies or nanobodies that traffic extracellular or transmembrane proteins to intracellular degradation machineries have been developed.

### Lysosome targeting receptor mediated extracellular or transmembrane protein degradation

**Fig. 2:**
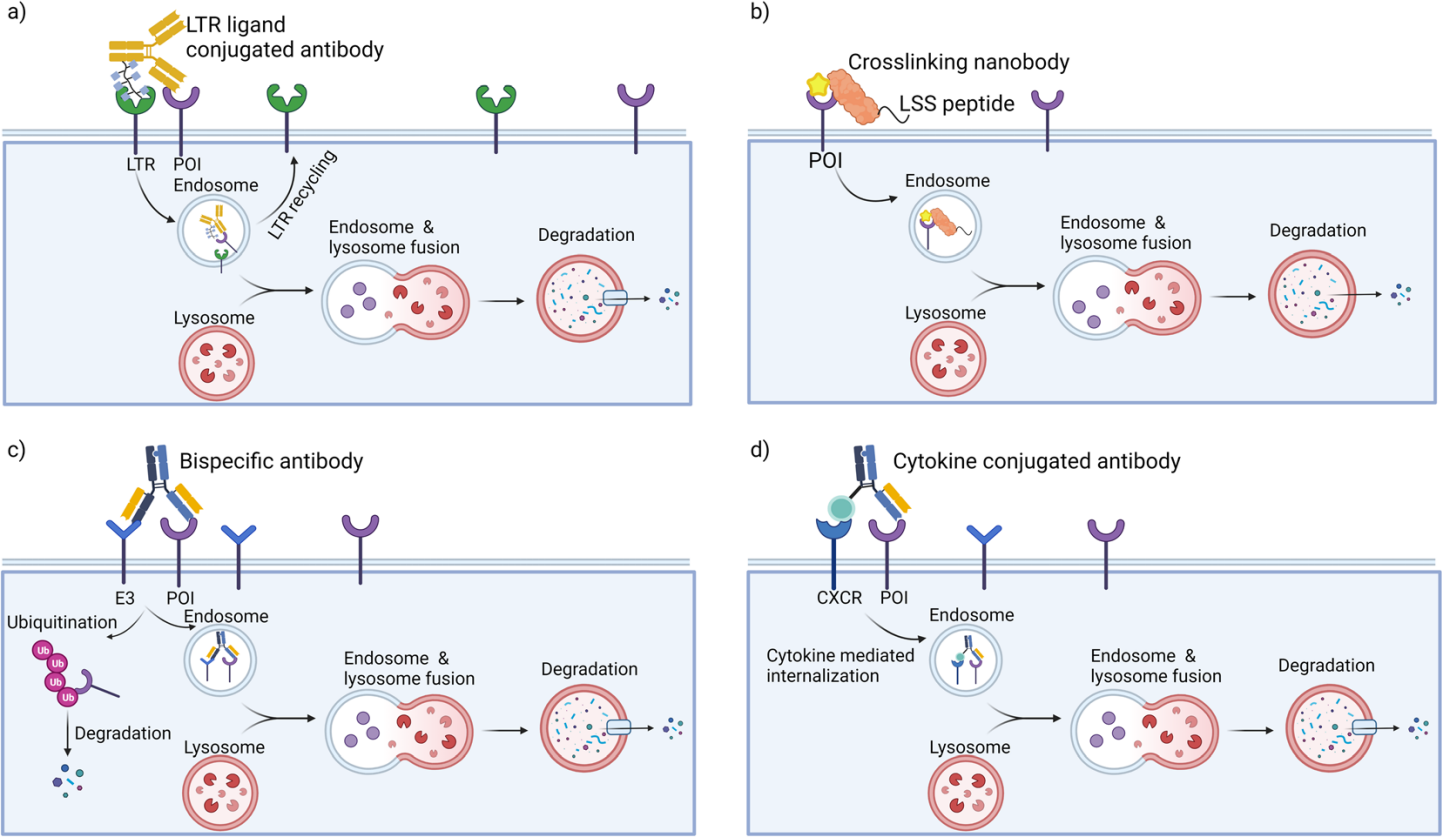
Targeted protein degradation platforms for transmembrane proteins. (a) LYTACs use antibodies modified with LTR binders to direct the transmembrane POI to the lysosome for degradation; LTR is recycled to the cell membrane. (b) Nanobodies functionalized with crosslinking moieties and lysosomal sequence to induce the lysosomal degradation of transmembrane proteins. (c) AbTACs use recombinant bispecific antibodies that engage transmembrane E3 ligases and proteins for lysosomal degradation. (d) KineTACs also employ recombinant bispecific antibodies that consist of one cytokine arm and one POI binding arm to induce internalization and degradation of transmembrane proteins.

Receptor mediated endocytosis provides a means to achieve specific protein degradation *via* the lysosomal pathway. LYTACs hijack the interaction of glycosylated proteins with lysosome-targeting receptors (LTRs) at the plasma membrane to shuttle transmembrane glycoproteins to the lysosome for degradation. LYTACs are constructed with two components: a POI binder and a LTR binder (Fig. 2a). The first generation of LYTACs harnessed the cation-independent mannose-6-phosphate receptor (CI-M6PR) as the LTR.^35^ By conjugating synthetic, nonhydrolyzable mannose-6-phosphonate (M6Pn) glycopeptides to antibodies, Ab-M6Pn conjugates were used to direct antibody targets to the lysosome *via* endocytosis. In the late endosome, the lower pH enabled dissociation of the targets and their procession to the lysosome for degradation, while the Ab-M6Pn was recycled back to the plasma membrane or Golgi. It was first demonstrated that conjugation of M6Pn ligand to biotin increased the internalization of the biotin binding protein NeutrAvidin in several cell lines. The strategy was then expanded to antibody binders, beginning with a polyclonal anti-mouse IgG non-specifically labeled with M6Pn glycopolypeptides on lysine residues to generate the IgG-M6Pn. Coincubation of mouse primary antibodies and IgG-M6Pn accelerated the degradation of a neurodegenerative disease target, ApoE4 and a cancer therapeutic target, CD71 (transferrin receptor-1). To further demonstrate the scope of LYTACs, direct M6Pn modification of the clinically approved antibodies cetuximab (Ctx) and Atezolizumab was performed. These two antibodies are known binders for cancer therapeutic targets epidermal growth factor receptor (EGFR) and programmed death ligand 1 (PD-L1), respectively. EGFR is a driver of cancer proliferation that harbors intrinsic tyrosine kinase function, with high abundance of EGFR uniquely found in some brain, lung, liver and other cancers.^66^ It performs multiple scaffolding functions regardless of inhibition of its receptor tyrosine kinase activity. PD-L1 is a transmembrane protein that is overexpressed on tumor cells; it binds to PD-1 receptors on activated T cells and mediates the inhibition of cytotoxic T cells; these deactivated T cells remain inhibited in the tumor microenvironment. The PD-1/PD-L1 pathway represents an adaptive immune resistance mechanism exerted by tumor cells for immune escape and has a high impact on the efficacy of cancer therapy.^67^ LYTACs created with Ctx and atezolizumab mediated selective loss of EGFR and PD-L1 by more than 70% within 48 h of treatment in several cell lines. The degradation was dependent on M6P binding and on lysosomal acidification. Additionally, a CRISPR interference screen uncovered an exocyst complex as a previously unidentified but essential component of biochemical pathway for CI-M6PR-mediated cargo internalization in cell lines. CI-M6PR is broadly expressed in most tissues, ensuring that the approach works in many settings.^35^

To achieve tissue specificity, second generation LYTACs were independently developed by the Bertozzi^36^ and Tang groups;^68^ these molecules engage the liver-specific ASGPR as the LTR (the Spiegel group has also targeted ASGPR with small molecule binders in a platform termed molecular degraders of extracellular proteins).^69^ ASGPR has exclusive expression in hepatocytes and functions to traffic glycoproteins bearing *N*-acetylgalactosamine (GalNAc) or galactose to the lysosome *via* clathrin-mediated endocytosis. Following internalization and endosomal acidification, ASGPR releases GalNAc and recycles back to the plasma membrane, while glycoproteins proceed to the lysosome for degradation. GalNAc-LYTACs efficiently ablated EGFR in HCC cells but not in cells lacking ASGPR. Whereas Ctx-M6Pn did not have cell-type selectivity, GalNAc-LYTACs are capable of cell-specific POI degradation. This could have implications for their development into therapeutics, as the ability to restrict POI loss to particular tissues could improve the efficiency of degraders.

LYTACs are capable of degrading both secreted and transmembrane proteins in several cell types. The flexible construct and modular design enable adaptation to binders that include antibodies, peptides, aptamers, and small molecules. The liver-specific GalNAc-LYTAC has presented the possibility of cell type-specific LYTACs. In depth studies to elucidate the spatial orientation required for LTR recognition, kinetics of LTR turnover, the rates of target trafficking, and sorting through the endocytic pathway will be critical for translational applications.

Inspired by LYTACs, GlueTAC^70^ was developed by fusing a nanobody with a lysosomal-sorting sequence so as to target transmembrane proteins (Fig. 2b). In this approach, the PD-L1 nanobody (Nb-PD-L1) was engineered to incorporate a fluorosulfate-l-tyrosine (FSY) for covalently labeling of POIs. By conjugating the covalent nanobody to a cell penetrating peptide consisting of a nona-d-arginine peptide and a lysosomal-sorting sequence (NPGY), the resultant GlueTAC led to the internalization and lysosomal degradation of more than 89% of PD-L1 and showed sustained T-cell activation and tumor growth inhibition. Without the FSY modification, only 32% of PD-L1 was degraded. The irreversible covalent binders have the potential to improve the degradation efficiency of POIs and to avoid off-target effects during endocytosis. However, specific screening to identify unnatural amino acid incorporation and modification sites is required for each target, which may hamper the broad application of GlueTACs.

### Bi-specific antibodies mediating transmembrane and extracellular protein degradation

To target transmembrane proteins, the Wells group developed fully recombinant bi-specific antibodies known as AbTACs, which bring the transmembrane E3 ligase RNF43 into close proximity with transmembrane POIs to mediate their degradation (Fig. 2c).^37^ Their work utilized phage display to identify a binder for the ectodomain of RNF43. To target PD-L1 specifically, AbTACs were constructed using a “knobs-into-holes” technique with two half-IgGs aimed at RNF43 and PD-L1, leading to a 63% degradation of PD-L1. In contrast to small molecules and LYTACs, the fully recombinant bi-specific IgG scaffold has favorable pharmacokinetic properties.

In normal cells, the expression level of RNF43 is highly constrained. However, hyperactivation of Wnt signaling in colorectal cancer results in an elevated expression of RNF43 and ZNRF3. This abnormally high expression level enabled scientists at Genentech to develop the colorectal cancer (CRC)-specific degraders by adopting bi-specific antibodies.^38^ A series of antibodies with picomolar to nanomolar affinities against the ectodomains of RNF43 and ZNRF3 were obtained. Focusing on the insulin growth factor 1 receptor (IGF1R), a receptor tyrosine kinase that mediates growth factor signaling in various tissues and cancers, ZNRF3*IGF1R bispecific antibodies showed clearance up to 80% with specificity for CRC both *in vitro* and *in vivo*. Mechanistic studies revealed that ZNRF3*IGF1R antibodies can engage both proteasomal and lysosomal degradative pathways in a catalytic manner. The CRC-specific degradation seen with ZNRF3*IGF1R was demonstrated to be applicable to other cell membrane targets (such as HER2 and PD-L1) and transmembrane E3 ubiquitin ligases.

Genetic optimization was used to investigate the structure–activity relationship (SAR) between different bi-specific antibody scaffolds and their degradation efficiencies, with the aim of understanding the properties necessary for efficient degradation.^38,71^ The study found that degradation efficiency is highly dependent on the specific epitopes of the E3 and the POI rather than binding affinity. However, the optimal epitope for an E3 ligase is not universal to all targets, as it varies due to the different complexes formed between POIs and E3 ligases. The flexibility, valency, and orientation of the binding arms in IgG scaffolds also affect degradation efficiency. Therefore, careful optimization of the epitope, affinity, orientation, format, and combination of antibodies is necessary to achieve efficient degradation.

The above-mentioned bi-specific antibodies rely on transmembrane E3 ligases and intracellular ubiquitin transfer, limiting their scope to transmembrane proteins and leaving the secreted proteome out of reach. To address this limitation, the Wells group developed a new technology called KineTACs, constructed with genetically encoded human bi-specific antibodies that contain a cytokine arm to target a cytokine receptor, and a target-binding arm for POIs (Fig. 2d).^39^ By focusing on CXCL12, they demonstrated that KineTACs can efficiently utilize CXCR7 internalization for lysosomal degradation. KineTACs are generalizable against other cytokines (*e.g.*, CXCL11, vMIPII, and IL-2) and have been used to degrade eight therapeutically relevant transmembrane and secreted proteins (PD-L1, HER-2, EGFR, and CDCP1, VEGF, TNF-α, CXCL11, and vMIPII). Compared to LYTACs and AbTACS, CXCL12-based KineTACs are exquisitely selective in degrading target proteins with minimal off-target effects, as revealed by proteomic analyses. However, KineTACs require the target protein to be internalized by a cytokine receptor, which may not be available or functional in all cell types or under all conditions. Additionally, the cytokine arm of KineTACs may activate signaling pathways and induce cytokine-related toxicities *in vivo*, which needs to be carefully evaluated during preclinical development.

## Protein-based PROTACs for targeting intracellular proteins

**Fig. 3:**
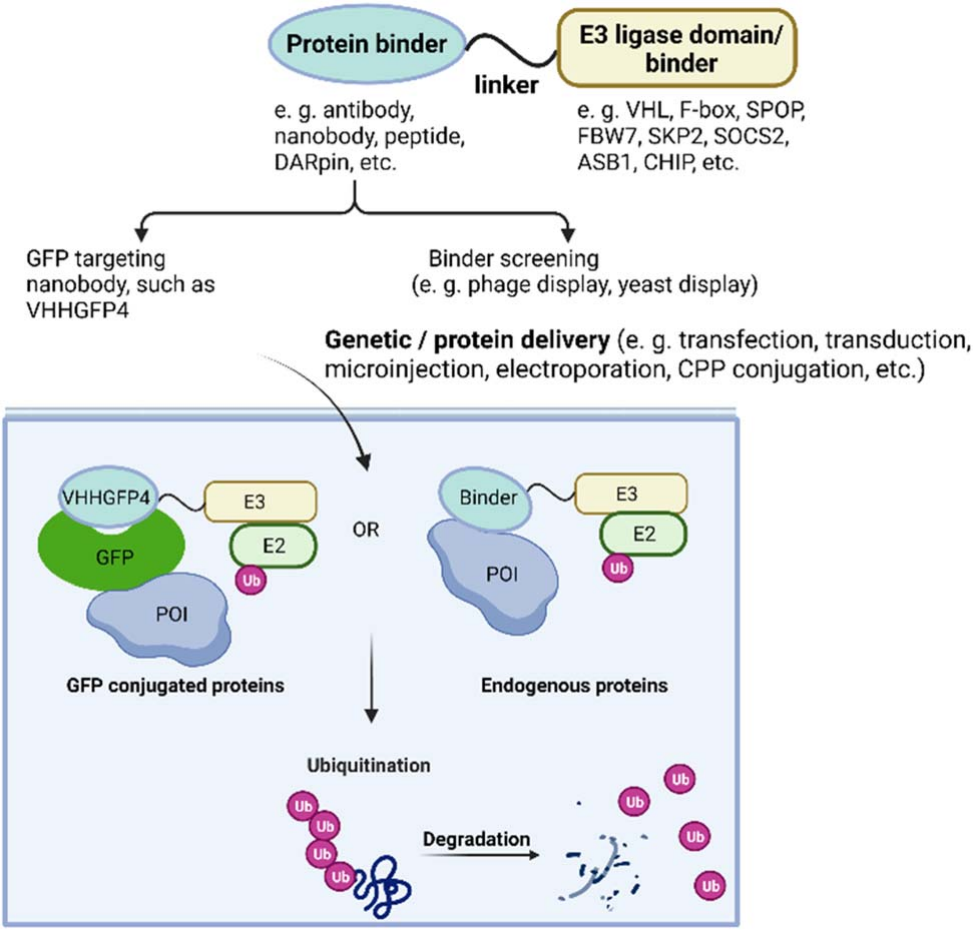
Overview of protein-based PROTAC design. With protein or gene delivery methods, protein-based PROTACs enter cells and engage with both POI and E3 ligase, leading to polyubiquitination subsequent degradation of the POI.

Ubiquitin-dependent proteolysis is a crucial pathway for regulating the levels of intracellular proteins as part of normal cellular maintenance processes. There are more than 600 E3 ligases encoded by the human genome,^72^ with each E3 ligase having a dedicated type of substrate.^73^ The substrate specificity of the UPS is determined by the substrate-recognition domains within the E3 ubiquitin ligase complexes. To manipulate intracellular E3 ligases, protein-based PROTAC strategies primarily focus on engineering substrate-specific nanobodies or other binding moieties within E3 ligases to redirect the UPS system towards non-substrate protein targets. In contrast to small-molecule PROTACs, which frequently use small molecule binders for cereblon (CRBN), VHL, MDM2, and cIAP in their designs, protein-based PROTAC platforms use a much broader range of E3 ligases. These platforms include, but are not limited to, ubiquibodies,^74^ TRIM21-nanobody fusion proteins,^75^ chimeric F-box fusion proteins,^76^ the affinity-directed protein missile system (AdPROM),^77^ and the antibody RING-mediated destruction (ARMeD)^78^ system. In this section, we provide a brief introduction to these technologies and focus on several examples that demonstrate their specificity in degrading protein isoforms (Fig. 3). Additionally, we highlight protein delivery methods, particularly the recent development of cell-permeant protein-based PROTACs for intracellular targets.

### Protein-based PROTACs for targeting green fluorescent protein (GFP)-tagged proteins

A series of pioneer protein-based PROTACs demonstrated their degradation abilities using intracellular POIs fused with GFP. In these designs, the substrate recognition module of the E3 ligases was replaced with a GFP binder such as the anti-GFP nanobody vhhGFP4. For instance, Affolter and colleagues developed the deGradeFP technique in 2012, which used the N-terminal domain of Slmb, a F-box protein identified in *Drosophila melanogaster*, fused to vhhGFP4 to induce the degradation of proteins with GFP fusion in both mammalian cells and Drosophila.^79^ In 2016, the Sapkota group engineered the AdPROM system by modifying VHL to target GFP-tagged proteins for proteolysis in mammalian cells.^80^

To assess the impact of different binders and E3 ligases on target degradation, the Partridge group characterized various E3 ligases and GFP binding scaffolds on GFP-tagged histone 2B.^81^ The tested E3 ligases included CUL1 ligases beta-transducin repeat containing protein (β-TRCP), F-box, and WD repeat domain-containing 7 (FBW7), and S-phase Kinase-associated Protein 2 (SKP2); CUL2 ligase VHL; CUL3 ligase speckle type BTB/POZ protein (SPOP); CUL5 ligases suppressor of cytokine signaling 2 (SOCS2), ankyrin repeat and SOCS box-containing 1 (ASB1); and U-box E3 ligase carboxy-terminus of Hsc70-interacting protein (CHIP). The results revealed that at least half of the E3 ligases tested led to more than 70% depletion of the POI. Additionally, a range of binding scaffolds such as peptides, nanobodies, designed ankyrin repeat proteins (DARPins), alpha repeat proteins, and monobodies, can successfully target GFP-tagged proteins. By replacing the GFP-binding domain with the 16-amino-acid Con1 peptide, which contains the conserved PIP sequence common to proliferating cell nuclear antigen (PCNA) binding partners, the protein-based PROTAC Con1-SPOP was developed and rapidly induced the degradation of PCNA upon doxycycline-induced expression. These discoveries indicate that protein-based PROTACs offer significant advantages in terms of linker and E3 ligase flexibility.

### Protein-based PROTACS incorporating specific binders to target intracellular protein isoforms

Protein-based PROTACs provide researchers with a high level of flexibility when selecting binders for degradation targets and E3 ligases, thereby enabling the degradation of “intractable” unmodified therapeutic targets (*i.e.*, proteins for which small molecule binders have been difficult to obtain). However, extending the protein-based PROTAC platform to endogenous proteins requires the identification of highly specific binders, which can be challenging due to high sequence similarity between homologs, variants, and isoforms of proteins. Here, we highlight successful examples of protein-based PROTACs that distinguish the target protein from closely related homologs.

The downstream signaling of RHO small GTPases is mediated by its ability to switch between guanosine diphosphate (GDP)-bound and guanosine triphosphate (GTP)-bound states. A phage display selection protocol was used to identify an intracellular single domain antibody (intrabody) that selectively targets only the active form of isoform RHOB-GTP, while avoiding other confirmations.^82^ To prevent cross-reactivity with isoforms RHOA and RHOC, pre-clearing steps were carried out in the presence of an excess of GDP-loaded wild-type RHOB and constitutively active GTP-bound RHOA and RHOC variants. The enriched pool of intrabodies against RHOB-GTP was then subcloned, and a direct cell-based degrader screening of F-box-intrabody fusions was performed. This screening method allowed the enriched intrabodies to show high specificity for RHOB-GTP and not for other conformations. In addition, the method enabled quick identification of the best combinations of F-box and intrabody. This study highlighted the benefits of protein-based PROTACs in comparison to small molecule PROTACs, particularly in terms of binder identification, specificity, and flexibility.

KRAS is a member of the Ras gene family, which encodes HRAS, NRAS and KRAS. RAS proteins are small G proteins with intrinsic GTPase activity, contributing to activation of downstream effectors involved in multiple pathways including apoptosis, proliferation and differentiation.^83,84^ KRAS mutations are some of the most prominent genetic alterations in human cancers, representing the major drivers of colorectal, lung, and pancreatic cancers. KRAS mutations occurs mainly at position 12, 13 or 61, with the most common being G12C (32.1%), G12D (23.4%), G12V (21.1%) and G12A (12.8%).^83^ Selectively targeting KRAS is challenging due to the high similarity of RAS isoforms, which share 82–90% amino acid sequence identity.^85^ Currently, small molecule inhibitors are only effective against the KRASG12C variant.^86^ In recent years, several studies have reported the use of protein-based PROTACs to target KRAS. One example of this is the use of the highly selective DARPin K19 to develop specific degraders for KRAS. DARPin K19 was identified previously through a phage display selection of a diverse DARPin library against KRASG12V.^87^ It has been reported that DARPin K19 specifically inhibits KRAS by binding to an allosteric site that includes the region around KRAS-specific residue histidine 95 at the helix α3/loop 7/helix α4 interface. While DARPin K19 does not distinguish between mutant and wild-type KRAS (KRASWT), it does not bind to NRAS and HRAS. Fusing DARPin K19 to the E3 ligase VHL created a KRAS-specific degrader that induces specific proteolysis of both variant and wild type KRAS.^88^ This is in contrast to the pan-RAS degrader, which employed a known pan-RAS intracellular single domain antibody (iDAb) that binds all three isoforms of the RAS family (KRAS, HRAS, and NRAS) and leads to degradation of all these proteins without selectivity. These results indicate that the specificity of protein-based PROTACs depends on the POI binders used in their construction. It is noteworthy that while the KRAS-specific degrader was able to eliminate both wild-type and variant KRAS, it only inhibited cell proliferation in cancer cell lines with the KRAS-variant present. In addition, KRAS degradation also mediated tumor regression in the KRAS mutant H358 mouse xenograft model of non-small cell lung cancer.

Another example of a protein-based PROTAC targeting KRAS mutations is the monobody 12VC1 reported by the Koide group.^89^ This monobody was identified through a combination of phage display and yeast display technologies. It selectively binds to activated forms of the KRAS G12C and G12V variants, showing up to 400-fold difference in affinity for variants over wild type. The crystal structure of 12VC1 bound to HRAS (G12C) with GTPγS reveals that 12VC1 recognizes the amino acid residue at position 12 through a shallow pocket. When expressed in cells, 12VC1 alone inhibits ERK activation and the proliferation of RAS-driven cancer cell lines containing KRAS (G12V) and (G12C). However, the VHL-12VC1 fusion provided more extended suppression of mutant RAS activity compared with inhibition alone. The higher potency of the degrader was attributed to its ability to engage multiple targets, which reduces the effective concentration required for occupancy-based inhibition. Notably, inhibition alone led to an eventual increase in the production of the RAS mutants in cells while selective degradation combats this feedback and highlights the potential of variant-selective RAS degraders as an effective therapeutic strategy.

In other work, the Partridge group^90^ explored a series of anti-RAS protein-based PROTACs using binders with different affinities, isoform specificities, and GTP/GDP-bound selectivity. By tagging the RAS protein with Nanoluc luciferase at its N-terminus, they monitored RAS degradation quantitatively. The outcomes of these degraders have demonstrated that the specificity of protein-based PROTAC mediated degradation is driven by precise interactions of the binders with POIs. One such degrader, K27-SPOP, uses DARPin K2 as a specific binder for GDP-bound RAS. Its use results in an increased degradation in the presence of AMG510, an inhibitor that promotes the inactive GDP-bound state of KRAS^G12C^. Interestingly, K27-SPOP showed different degradation efficiencies for wild type and various KRAS variants, and the research uncovered that not all variants are locked in the active, GTP-bound state; there is relative prevalence of the “off-GDP-state” in the cellular context.

### Protein delivery strategies for protein-based PROTACs

While protein-based PROTACs present exciting opportunities for the targeted degradation of intracellular proteins, one of the major hurdles for *in vivo* use, especially for clinical translation, is delivery of the degrader into cells. Currently, gene delivery approaches including viral transduction^75,91^ and transient transfection,^81^ as well as physical strategies (electroporation^92^ and microinjection)^93^ have been widely used to demonstrate the *in vitro* efficacy of protein-based PROTACs. To date, the efficacy of protein-based PROTACs *in vivo* has only been demonstrated through limited proof-of-concept examples^88,89,94^ that used microinjection or promoter inducible transduction. Given that these methods are unsuitable for clinical applications, further development of delivery strategies to ensure safe and efficient *in vivo* delivery could advance the translation of protein-based degraders.

### Genetic approaches to deliver protein-based PROTACs

One strategy to deliver protein-based PROTACs into cells uses genetic approaches. The delivery methods are typically classified as transfection or transduction approaches.^95,96^ With transfection, cationic polysaccharides, synthetic polymers, lipids, and peptides are used as delivery vehicles. These positively charged reagents encapsulate negatively charged nucleic acids, which then form a complex that associates with the negatively charged cell membrane. Although the exact mechanisms by which these complexes enter cells remain unknown, it is generally believed to be related to endocytosis or phagocytosis. These entry methods are advantageous because of their relatively low cytotoxicity and controlled immunogenicity.^95,96^ Moreover, they do not require mutagenesis and have no size limitation on the packaged nucleic acid. However, they often suffer from low delivery efficiencies and varying levels of endosomal escape. With transduction, viruses, such as lentivirus and adeno-associated virus (AAV) are employed for delivery.^97^ Chemical transfection efficiency varies depending on nucleic acid/chemical reagent ratio, solution pH, and the types of cell being transfected, while the advantages of transduction relate to efficiency, sustainable transgene expression, and the potential of achieving tissue- and cell-selectivity.^97^ Many protein-based PROTACs relied on transfection/transduction to introduce the degrader DNA into target cells and to assess degradation efficiencies.^79,81,82,98^ In a clinically relevant viral gene delivery strategy, Gallardo *et al.* used AAV to deliver anti-tau protein degraders *in vivo*.^99^ However, severe limitations that include immunogenicity, cytotoxicity and limited payload versatility, remain. As an example, the size of the *trans*-gene is limited to ∼4 kb for AAV to efficiently facilitate its delivery. Whereas lentivirus has been used for protein-based PROTAC gene delivery,^91^ it does integrate into the chromosome randomly and may thereby disrupt tumor suppressor genes, activate oncogenes, or interrupt essential genes. These disadvantages have negatively impacted the application of viral delivery systems, and thus protein-based PROTACs, in clinical studies.

### Physical approaches to deliver protein-based PROTACs

An alternative method to deliver protein-based PROTACs is by introducing nucleic acids or pure proteins into cells through microinjection and electroporation. For example, with microinjection, antibodies and Trim-21 proteins were directly introduced into individual cells to enable Trim-Away mediated degradation.^75^ Hwang and colleagues injected degrader mRNA into the embryo zebrafish to deplete Hmga2-Citrine.^100^ However, microinjection is a labor-intensive and skilled approach that can only be used on a small scale, and its potential for clinical application is limited. On the other hand, electroporation creates pores in the cell membrane using a short electrical pulse, allowing proteins to pass into the cell. This method is easy, fast, and enables a large number of cells to be transfected in a short time. For example, Clift *et al.* used electroporation to confirm the efficacy of Trim-Away in bulk populations of cells and to adapt the method for larger-scale assays.^75,101^ Similarly, Ibrahim *et al.* electroporated degrader proteins into cells to test the ARMeD system and found that electroporation of pure proteins resulted in a much higher degradation rate (within minutes) than that of expression (within hours).^78^ The accelerated rate of degradation is attributed to the timing of protein production, with transfection of mRNA or DNA requiring a longer time to produce sufficient amounts of degrader proteins. Zeng *et al.* also used electroporation to deliver antibodies for a Trim-Away application.^102^ An advantage of electroporation is that it avoids accumulation in endosomes or lysosomes, and maintains cell viability for further studies, making this method particularly useful for delivering protein-based degraders. However, it is largely relegated to *in vitro* applications.

### Cell-permeant protein-based PROTACs for targeting intracellular proteins

An alternative approach to overcome the translational limitations of the above-mentioned delivery methods is the development of cell-permeant protein-based PROTACs. This is done by conjugating or appending cell penetrating peptides (CPPs) to the protein-based degrader.^103^ CPPs are short amino acids that can translocate cargo across the plasma membrane and facilitate their delivery to the cytoplasm. Since the mid-1990s, thousands of putative CPPs such as supercharged proteins, cyclic peptides, hydrocarbon stapled peptides, and miniature proteins, have been identified.^103,104^ Although their mechanisms of delivery are not all fully understood, it is believed that the positively charged residues (either Lys or Arg) in most CPPs form electrostatic interactions with sulfated proteoglycans on the cell membrane and induce endocytosis. Developing a universal CPP that can deliver diverse protein cargos without disrupting cell function would offer a more efficient platform for further translational studies.

Very recently, our group created a protein-based PROTAC to deplete endogenous BCL11A, a transcription factor validated as a therapeutic target for the hemoglobin disorders sickle cell disease and β-thalassemia.^40^ Our design fused a nanobody binder specific for BCL11A to the cell-permeant miniature protein ZF5.3 (ref. 105 and 106) and to tSPOP, the catalytic domain of the E3 adapter protein SPOP. This protein-based PROTAC depleted cellular BCL11A in differentiated primary erythroid precursor cells and induced the expression of fetal hemoglobin (HbF).

As with other transcription factors, developing PROTACs for BCL11A is difficult because of BCL11A's intrinsic structural disorder and its close similarity to a paralog, BCL11B.^107^ Despite being largely unstructured, BCL11A contains several well-ordered regions, including a CCHC zinc finger (ZnF0) and six C2H2 zinc fingers (ZnF1–6). The ZnF0 domain is involved in self-association of BCL11A,^108^ while ZnF2–6 are critical for its function in suppressing HbF expression.^109^ Targeting these domains with specific binders holds great potential for modulating the activity of BCL11A and its role in regulating HbF expression. However, the zinc finger regions of BCL11A and its paralog BCL11B share high sequence similarity, which may lead to binders recognizing both proteins. To address this, we created fragments of the well-ordered ZnF domains but included “extended”, presumptive disordered regions where the sequence differs between paralogs. For example, in targeting the extended ZnF23 (exZnF23) domain, we used yeast display to identify nanobody binders that preferentially bind to BCL11A over BCL11B; negative selection with ZnF23 domain eliminated binders that recognize BCL11B. This selection step was followed by error prone mutagenesis to identify binders with improved affinities.

With these highly selective nanobody binders available, we proceeded to deplete BCL11A. The nanobody used in this design, 2D9, binds to ZnF23 of BCL11A with an equilibrium dissociation constant (*K*_d_) of approximately 300 nM. Functionalization of 2D9 for cell penetration was achieved by appending the cell-permeant miniature protein ZF5.3 to 2D9, as ZF5.3 was reported to show superior cytosolic delivery of protein cargos than most CPPs. The fusion of ZF5.3 to 2D9 did not significantly impact its binding to endogenous BCL11A, and ZF5.3 mediated the cellular uptake of 2D9 in a dose- and time-dependent manner. The fusion protein, ZF5.3-2D9, was predominantly located in the nucleus of HUDEP-2 cells, a cell line resembling adult erythroid precursor cells.

To first verify the utility of 2D9 as a POI binder for protein-based PROTAC development, plasmids of 2D9 fused to the Fc domain of immunoglobin G1 or Trim 21 were constructed. Both 2D9-Fc and 2D9-Trim21, introduced through transduction, successfully degraded BCL11A in HUDEP-2 cells. Furthermore, neither induced loss of the paralog BCL11B, indicating that 2D9 exhibits high specificity for its BCL11A antigen. Encouraged by this, cell-permeant degraders were developed by appending the substrate domain of SPOP or RNF4 to ZF5.3-2D9. The degrader ZF5.3-2D9-tSPOP demonstrated up to 70% loss of BCL11A 12 h after treatment; this loss was sustained for at least 72 h. Treatment in the presence of a proteasome inhibitor, MG-132, prevented BCL11A degradation and confirmed that protein loss was proteasome-dependent.

Reducing BCL11A expression has been shown to alleviate symptoms in individuals with hemoglobin disorders *via* reactivation of HbF expression.^110,111^ However, only the genetic approaches clustered regularly interspaced short palindromic repeats (CRISPR) – Cas9 and RNA interference are used in patients.^112^ The sustained BCL11A degradation observed upon protein-based PROTAC treatment in HUDEP-2 cells prompted an investigation into the effect of ZF5.3-2D9-tSPOP on HbF induction in differenced HUDEP-2 and primary human hematopoietic stem cells (CD34^+^ cells). During erythropoiesis, hemoglobin (Hb) synthesis increases from low levels in early progenitors to significantly higher amounts in mature enucleated erythrocytes. Consistent with its functional role as the Hb regulator, BCL11A levels also change during differentiation. As such, the timing of degrader delivery is critical. In both HUDEP-2 and CD34^+^ cells, significant HbF induction was observed during differentiation after treatment with the protein-based PROTAC. The treatment with ZF5.3-2D9-tSPOP had no impact on erythropoiesis of CD34^+^ cells, as demonstrated by comparable expression levels of differentiation surface markers CD235a and CD36 in treated and control cells. However, treated CD34^+^ cells exhibited slower proliferation compared to control, possibly due to BCL11A loss – this observation is consistent with studies using CRISPR-Cas9 deletion and shRNA silencing of BCL11A.^113^ The observed loss of BCL11A and induction of HbF were directly related to BCL11A engagement with 2D9, as omission or replacement of 2D9 in the protein-based PROTAC design prevented these effects.

Compared with genetic approaches targeting BCL11A, the cell-permeant protein-based PROTAC strategy provides a reversible, temporal way of modulating BCL11A. Furthermore, the success of BCL11A degradation and HbF induction in primary hematopoietic stem cells is supportive of enhanced intracellular stability and low-toxicity of protein-based degraders.

**Fig. 4:**
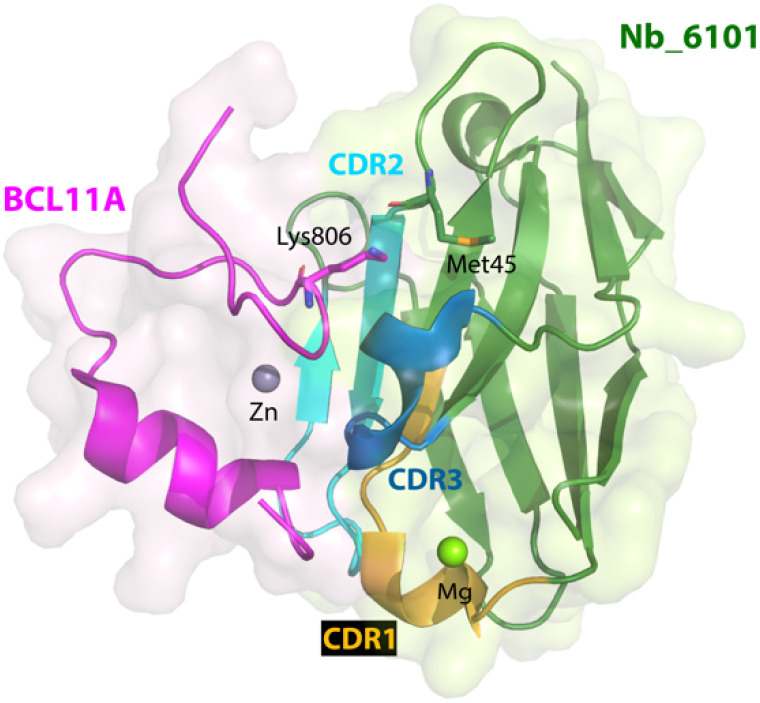
X-ray crystallographic structure of nanobody Nb_6101 in complex with ZnF6 of BCL11A (PDB 8DTN). Nb_6101 is shown in green and ZnF6 in magenta. Methionine 45 in Nb_6101 close to Lysine 806 in ZnF6 could be replaced to increase binding affinity.

In parallel with this study, nanobodies targeting another fragment of BCL11A were also obtained.^114^ In contrast to exZnF23, the ZnF6 fragment is well-ordered but also distinct from the paralog BCL11B. Using the same strategy for ligand discovery, an initial nanobody binder for ZnF6 was evolved from a ligand with *K*_d_ of 2.75 μM to one with a *K*_d_ < 700 nM *via* error prone mutagenesis. A crystallographic structure of the nanobody-BCL11A ZnF6 complex (Nb_6101-ZnF6) revealed that ZnF6 interacts with complementarity determining regions 2 and 3 of the nanobody. Site No. 45 on the nanobody Nb_6101 was shown to be critical because of its interaction with Lys806 on ZnF6; mutagenesis to convert Met45 of Nb_6101 (Fig. 4) to either an aspartic acid, a glutamic acid, a serine, or a threonine allowed for the formation of a hydrogen bond with Lys806, which improved binding affinity by approximately 6-fold. Computational modeling provided additional insights to enable the optimization of regions near site No. 45, and subsequent mutagenesis produced nanobody binders with significantly improved affinities (*K*_d_ ∼ 20 nM). The approaches used here to guide the evolution of nanobodies led to ∼100-fold enhancement of their affinities for the target. With these evolved high-affinity binders, conjugation to Trim21 also led to the Trim-Away mediated degradation of BCL11A but not BCL11B. These two studies highlight the differences of targeting a well-ordered protein (ZnF6) *versus* one with structural disorder (exZnF23) but show that the latter is feasible.

**Fig. 5:**
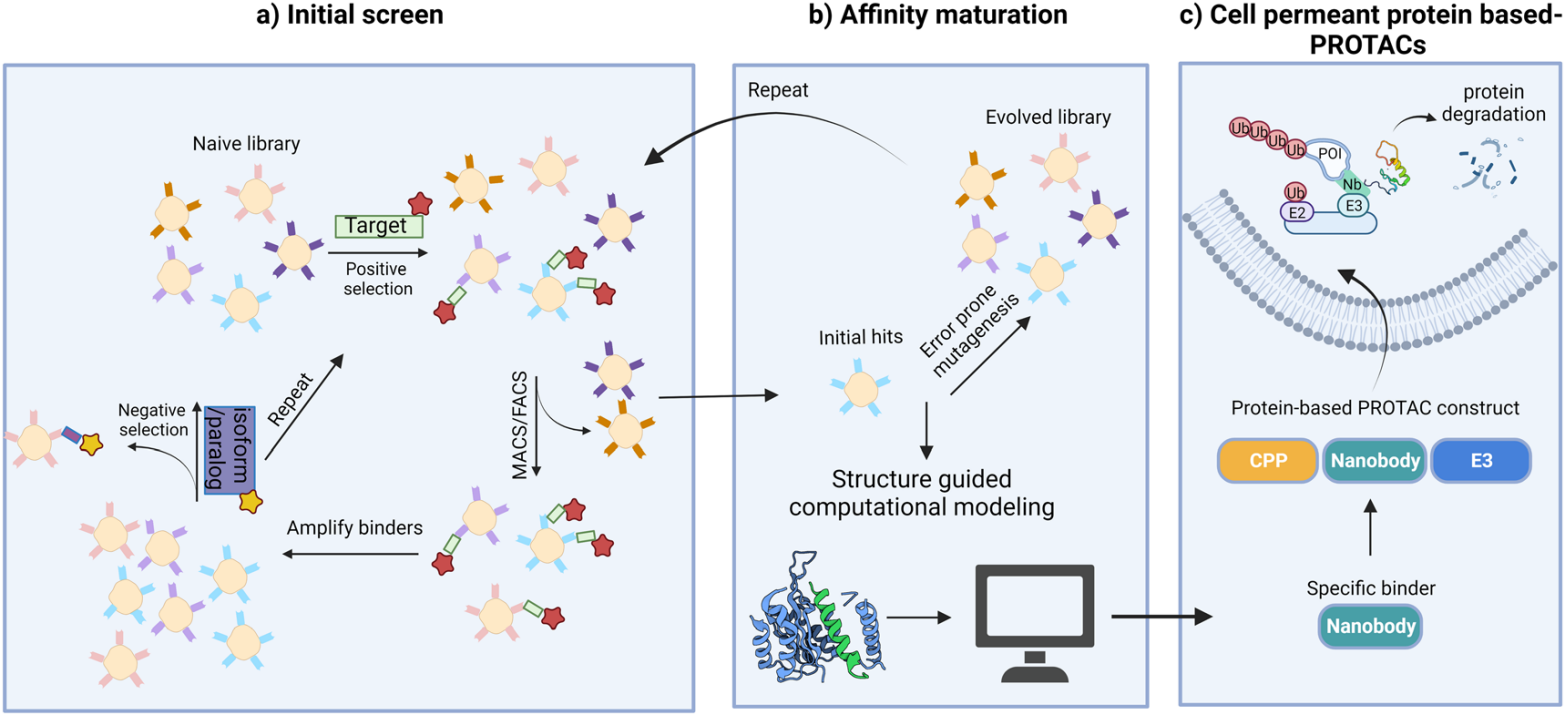
Strategies to target “undruggable” soluble proteins with cell-permeant protein-based PROTACs. (a) Screen to identify initial nanobody binders; (b) mutagenesis or structure guided computational modeling to improve binding affinities; (c) functionalization of nanobody binders for cell penetrance and E3 ligase recruitment leads to POI degradation.

Our strategy of systemic ligand identification, cell penetrance functionalization, and protein-based PROTAC construction provides a new possibility for the targeted degradation of other difficult-to-drug proteins (Fig. 5). Additional studies to understand the details of protein translocation, ligand orientation, and structure–activity relations of binders and E3 ligases are critical for improving the degradation efficiencies and broadening the applications of protein-based PROTACs. Finally, strategies to achieve cell specificity *in vivo* will be crucial for future clinical applications of protein-based degraders.

## Conclusions and outlook

TPD has had a tremendously positive impact on the fields of chemical biology, cancer biology, immunobiology, and drug discovery.^115^ Studies on protein degradation pathways have yielded valuable insights into the development of diverse TPD technologies, which all function by exploiting endogenous systems. The development of protein-based PROTACs has revealed exciting possibilities for targeting a wide range of proteins, including membrane proteins, secreted proteins, and highly disordered proteins. Additionally, a wider array of E3 ligases has been employed, providing higher flexibility compared to small molecule degraders. The success in the past two decades and recent developments in both academia and industry suggest that TPD could become a key modality for developing therapeutics.

Despite this, the widespread clinical applications of protein-based PROTACs face significant challenges. Although protein-based PROTACs offer greater flexibility in terms of binders and E3 ligases selection, it is important to note that some examples have failed for unknown reasons,^71,81^ and that the rules for achieving efficient degradation of a particular POI are still not clear. The degradation appears to be influenced more by the epitope of the POI/paratope of the binder than by affinity. Additionally, synergy of ligand, target POI, and E3 ligase is critical for efficient degradation, but molecular control of that synergy is yet to be understood. As such, achieving successful and efficient POI degradation requires careful optimization of ligand affinity, specificity, and orientation, and may require the screening of multiple E3 ligases. The direct screening of protein-based degraders using combinatorial domain libraries can also be considered.

Finally, the application of protein-based PROTACs *in vivo* is challenging due to limitations in achieving selective cell type delivery. However, the recent development of protein delivery methods such as the bacterial contractile injection system,^116^ the botulinum neurotoxin platform,^117^ and functionalized nanoparticles^118^ provide exciting possibilities that might accelerate the clinical applications of protein-based PROTACs. It is our hope that efforts from our group directed at expanding target scope to intrinsically disordered protein regions, performing in-depth studies of structure activity relationships, achieving tissue- and organ-specific delivery, coupled with investigations on pharmacokinetics, pharmacodynamics, off-target side effects and toxicities, will enable the translational applications of protein-based PROTACs.

## Author contributions

F. S. and L. M. K. D. conceptualized, wrote, and edited the Perspective.

## Conflicts of interest

There are no conflicts to declare.

## Supplementary Material
